# Designer Oncolytic Adenovirus: Coming of Age

**DOI:** 10.3390/cancers10060201

**Published:** 2018-06-14

**Authors:** Alexander T. Baker, Carmen Aguirre-Hernández, Gunnel Halldén, Alan L. Parker

**Affiliations:** 1 Division of Cancer and Genetics, Cardiff University School of Medicine, Cardiff CF14 4XN, UK; BakerAT@Cardiff.ac.uk; 2 Centre for Molecular Oncology, Barts Cancer Institute, Queen Mary University of London, London EC1M 6BQ, UK; etheluin@gmail.com (C.A.-H.); g.hallden@qmul.ac.uk (G.H.)

**Keywords:** adenovirus, oncolytic, targeting, virotherapy, cancer, αvβ6 integrin, immunotherapy, tropism

## Abstract

The licensing of talimogene laherparepvec (T-Vec) represented a landmark moment for oncolytic virotherapy, since it provided unequivocal evidence for the long-touted potential of genetically modified replicating viruses as anti-cancer agents. Whilst T-Vec is promising as a locally delivered virotherapy, especially in combination with immune-checkpoint inhibitors, the quest continues for a virus capable of specific tumour cell killing via systemic administration. One candidate is oncolytic adenovirus (Ad); it’s double stranded DNA genome is easily manipulated and a wide range of strategies and technologies have been employed to empower the vector with improved pharmacokinetics and tumour targeting ability. As well characterised clinical and experimental agents, we have detailed knowledge of adenoviruses’ mechanisms of pathogenicity, supported by detailed virological studies and in vivo interactions. In this review we highlight the strides made in the engineering of bespoke adenoviral vectors to specifically infect, replicate within, and destroy tumour cells. We discuss how mutations in genes regulating adenoviral replication after cell entry can be used to restrict replication to the tumour, and summarise how detailed knowledge of viral capsid interactions enable rational modification to eliminate native tropisms, and simultaneously promote active uptake by cancerous tissues. We argue that these designer-viruses, exploiting the viruses natural mechanisms and regulated at every level of replication, represent the ideal platforms for local overexpression of therapeutic transgenes such as immunomodulatory agents. Where T-Vec has paved the way, Ad-based vectors now follow. The era of designer oncolytic virotherapies looks decidedly as though it will soon become a reality.

## 1. Introduction

Once described by Peter Medawar (recipient of the 1960 Nobel prize for the discovery of immunological tolerance) as “*a piece of bad news wrapped up in protein*” [1], oncolytic viruses are beginning to emerge as clinically useful agents in the cancer arena. As long ago as 1953, viruses were reported to selectively kill cancer cells; Koprowski et al. concluded that “*Studies on the specificity of such a reaction for a given virus […] may facilitate an understanding of the mechanism of selective cell destruction*”, and in so doing, predicted what is now a global research effort to harness viruses as cancer therapeutics [2]. A wide range of different viruses are now under investigation as cancer therapeutics, ranging from wildtype small RNA viruses like Respiratory Enteric Orphan virus (Reovirus), to intricately engineered large DNA viruses such as herpes simplex [3,4].

Few virotherapies have seen as much development as those based on adenovirus (Ad). These non-enveloped, double stranded DNA viruses are generating increasing interest as therapeutic vectors for cancer owing to their low levels of pathogenicity and relative ease of manipulation. A search of clinicaltrials.gov shows in excess of 180 clinical trials utilising adenoviruses, in some form, as a cancer therapeutic at time of writing [5]. Whether the therapeutic agent is designed as a vaccine, gene therapy, or oncolytic virus, all are dependent on our ability to engineer these viruses and manipulate their natural tropisms to ablate pathogenicity and achieve therapeutic benefit.

**Figure 1:**
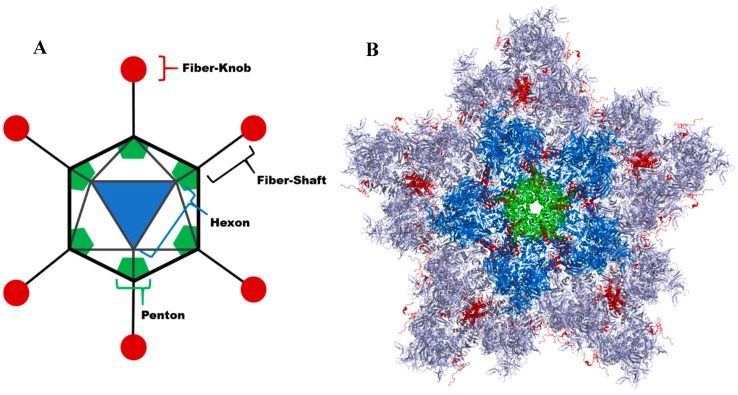
**Adenovirus Structure.** Cartoon view of adenovirus, highlighting the major capsid proteins as labelled (**A**). Structural view of an adenovirus vertex modelled from CryoEM structure (PDB: 6B1T) showing penton (green) with hexons (dark and light blue) and minor capsid proteins (red) (**B**).

This engineering is based on intricate understanding of adenovirus virology. These non-enveloped viruses possess an icosahedral capsid comprised of 3 major, and 4 minor, capsid proteins described in Figure 1. The hexon is the most abundant capsid protein, comprising the 20 facets of the dodecahedral viral capsid. At each vertex, shown in Figure 1, there is a penton base with the fiber protein projecting from it. These proteins influence secondary and primary viral tropisms, respectively (Figure 1). An in depth review of adenoviral structure can be found in the review by Russell [6].

Canonically there are 57 adenoviral serotypes divided amongst seven species—A-G—as determined by serological testing for neutralising antibody activity, though the true diversity may be much greater [7]. Phylogenetic analysis of the species D adenoviruses (the largest of the human adenoviral species) reveals homologous recombination between hypervariable regions in each of the major capsid proteins and in every member of this subtype. In turn, there will be a direct impact on capsid structure and thus immune recognition [8]. The authors of this study estimate that serology testing (the standard on which the canonical 57 serotypes is based) can only identify 53% of 38 fully characterised species D adenoviruses unambiguously. An alternative taxonomic proposal attempts to reflect the genetic variation observed in the ≈35 Kbp linear dsDNA genomes of isolated adenoviruses, yielding 86 candidate serotypes as of November 2017 [9,10].

Oncolytic adenoviruses must be capable of efficiently and selectively destroying cancerous cells and avoid damaging healthy tissues. This can be achieved through selective replication within cancer cells, a strategy important for virotherapies. Since the process of replication within the tumour microenvironment allows amplification of the therapeutic at the point of need, and the process of oncolysis (the “bursting” of virus filled tumour cells) is innately immunogenic, it facilitates a host-anti-tumour response. There are many means of achieving oncolysis, but most strategies rely upon the lytic properties of virotherapies, or encoding transgenes within the viral genome to induce necrosis or apoptosis, both of which serve to stimulate recruitment of immune cells to the tumour site. The review by Twumasi-Boateng et al. discusses oncolytic virotherapies as immunotherapies in detail and summaries the ongoing clinical trials in this area [11].

Some of the most significant oncolytic strategies are based around subtle mutations with early adenoviral genes to enable selective replication within cancerous cells whilst sparing non-transformed cells. Below we give further insights into the mechanistic basis of this means of selectivity.

Numerous attempts have been made, and are ongoing, to retarget adenovirus infection to cancer cells using chemical modification or adaptor proteins [12,13,14,15,16,17,18,19,20]. Such methodologies may be potentially hindered by the fact that the modifications are non-heritable and thus cannot be transferred to progeny virions. In the case of modifications conferring enhanced cell killing this means that progeny virions will have reduced oncolytic properties. Furthermore, modifications which target the therapy will also be lost raising the possibility that progeny virions may cause unwanted off target infection, with subsequent toxicities. While these problems can be prevented by the use of replication deficient Adenoviruses these vectors then lose a major advantage of oncolytic virotherapies: the capacity to self-amplify at the point of need [21,22,23].

This potential limitation can be overcome by incorporating modifications at the genetic level through manipulation of the viral genome, allowing the changes to be conferred to progeny through the normal viral replication cycle. Adenoviral genomes can be efficiently manipulated using recombineering technology [24]. This technique utilises homologous recombination to “capture” the adenovirus genome in a circular vectorised format, a Bacterial Artificial Chromosome (BAC), which can be maintained in *E. coli*. The vectorised gDNA (genomic DNA) can then be modified through further rounds of homologous recombination using a selectable marker cassette as an intermediary, enabling efficient production of mutant adenoviruses which can be rescued by transfection of the BAC into mammalian cells.

In this review we will discuss the diverse types of genetic modifications utilised to generate tumour specific oncolytic viruses. Initially, we will describe how viral cell killing can be regulated post-entry through transcriptional control and manipulation of the early viral proteins, before discussing how to prevent the natural viral tropisms from driving off target infection, and how these observations can guide the rational design of targeted viral particles to specific cell types through capsid engineering. Whilst there is also ongoing development of so-called “xeno-Ads”, adenoviruses with non-human hosts, this review will focus on the most clinically advanced and therapeutically relevant adenoviruses: those with human tropisms.

## 2. Replication-Selective Adenoviruses

To date, the majority of clinically evaluated mutants have been generated from species C serotypes 5 and 2, although, recently a species B chimera of serotype 3 and 11 has entered early phase clinical trials [25]. The most common strategy to generate replication-selective oncolytic adenoviral mutants is to delete viral genes that are essential for replication in normal cells, but are complemented in cancer cells with altered cell cycle, DNA damage-repair and cell death mechanisms [26]. Alternatively, tumour-selective promoters including androgen receptor response elements or telomerase promoters have been inserted to regulate early viral gene expression [27,28]. 

The first oncolytic adenovirus to enter clinical trials, *dl*1520 (named Onyx-015), was generated by deleting the viral E1B55K protein. Onyx-015 and the mutant H101 (both E1B55K- and E3B-deleted) are examples of replication-selective viruses carrying the E1B55K-deletion [29,30,31,32,33,34]. During the early stages of viral infection, E1B55K binds to cellular p53 and promotes G1/S transition in the presence of viral DNA and damaged host cell DNA. Expression of E1B55K is vital for adenovirus replication in normal cells, while in most cancers, p53 is non-functional through direct or indirect mutations in proteins regulating the p53 pathway [35]. Onyx-015 was demonstrated to replicate exclusively in cancer cells, although replication appeared to be independent of p53 status [36]. While safety of Onyx-015 and H101 has been demonstrated in numerous clinical trials, efficacy has been consistently disappointing because of attenuated viral replication and spread. Reasons for the poor efficacy was later revealed to be caused by defective nuclear export of viral mRNAs, a function directly controlled by E1B55K-binding to E4orf6. In addition, E1B55K inhibits cellular protein synthesis by facilitating export of the viral L4 100K to the cytoplasm to prevent activation of the elF-4F cellular factor, which is responsible for the translation of cellular mRNA [37]. Therefore, viruses lacking E1B55K replicate poorly also in the majority of cancer cells, contributing to the reported modest clinical outcomes [37]. Currently, trials with Onyx-015 have been discontinued while H101 has been approved for nasopharyngeal carcinomas in combination with chemotherapy by the Chinese State Food and Drug Administration (SFDA) [38]. 

**Figure 2:**
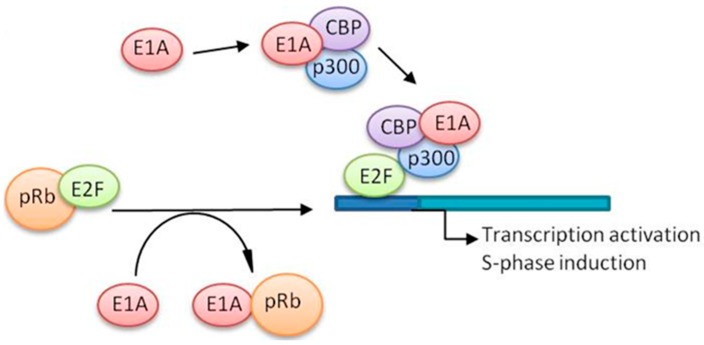
**Schematic representation of E1A-mediated regulation of the cell cycle.** The E1A adenoviral protein promotes S-phase induction by interacting with pRB and p300.

Due to the attenuated replication with E1B55K-deleted mutants, the next generation of oncolytic adenoviruses harboured small specific deletions in the E1A gene to retain viral potency while still being tumour-selective. Expression of E1A is an absolute requirement for viral replication through its binding to the retinoblastoma (pRb) protein, which releases E2F and induces S-phase entry, enabling the virus to exploit the cellular DNA and protein synthesis mechanisms (Figure 2) [39]. The E1A gene contains four conserved regions (CR1, CR2, CR3, and CR4) each with specific functions, for example, E1ACR1 binds to p300 and E1ACR2 to pRb. The cellular transcription factor E2F is normally repressed by the retinoblastoma protein (pRb) or its family members p130 or p107 [40]. When the small (24 amino acids) E1ACR2 domain binds to the pRb family of proteins, E2F is released and free to induce S-phase. E1ACR2-deleted mutants selectively replicate in cancer cells with deregulated cell cycle control (e.g., pRb-p16 pathway), but not in normal cells with intact growth control. The first oncolytic mutants with E1ACR2 deletions were *dl*922-947 and AdΔ24 [41,42]. Both mutants were demonstrated to potently and selectively replicate in preclinical cancer models to significantly higher levels than Onyx-015. 

To date, numerous E1ACR2-deleted mutants have been optimised for targeting of solid tumours. For example, the integrin retargeted Delta-24-RGD (DNX-2401) was reported to replicate and spread in recurrent malignant gliomas after local administration, prolonging survival in 20% of patients in a Phase I trial [43]. DNX-2401 is currently evaluated in two additional trials for patients affected by glioblastoma and glioma (NCT02798406; NCT03178032). The chimeric mutant Ad5/3-∆24 expressing Granulocyte-Macrophage Colony-Stimulating Factor (GM-CSF), Oncos-102 showed promising results in preclinical hamster models and in patients with solid tumours [44]. Currently, Oncos-102 is evaluated in several Phase I and II trials in combination with chemotherapy (NCT03514836; NCT02963831; NCT03003676; NCT02879669). In ICOVIR-7, the E1ACR2-deletion was combined with an E2F-promoter controlling E1A expression to mediate self-activation in tumour cells [45]. ICOVIR-7 was later armed with various transgenes and an RGD-4C integrin-targeting sequence (e.g., Ad5/3-Cox2L-D24), and has been evaluated in glioma patients with evidence of tumour reduction [46]. An improved version of this mutant, named VCN-01, had a putative heparin sulphate glycosaminoglycan (HSG)-binding site (KKTK) within the fiber shaft replaced by an RGDK motif, discussed later in this review [47]. This retargeted virus showed decreased liver transduction and increased tumour targeting in murine models. Furthermore, the human glycosylphosphatidylinositol-anchored enzyme PH20 hyaluronidase was inserted into the VCN-01 genome to promote viral penetration of the dense tumour stroma and to increase intra-tumoural spread, processes that may facilitate access by drugs and immune cells. Two phase I trials are underway with VCN-01 in combination with gemcitabine or Abraxane^®^, targeting patients with unresectable treatment-resistant pancreatic ductal adenocarcinomas (PDAC) (NCT02045589; NCT02045602).

In contrast to the prototype virus, Onyx-015 many E1ACR2-deleted mutants have the immunomodulatory E3gp19K gene deleted and retain the E3B genes. It has been established that deletion of E3gp19K enables antigen presentation through major histocompatibility complex (MHC) class I expression in the cell membrane of infected cells, which contribute to activation of T-cells and immune responses to cancer cell antigens. Retention of E3B genes was demonstrated to prevent premature elimination of virus by macrophages and enhance replication in tumours. A different approach was reported when generating the E1ACR2 deleted and RGD-integrin targeted virus ORCA-010 [48]. A single base mutation (T 29183) was incorporated in the E3gp19K protein that produced a protein (E3gp19K-T1; K445A) lacking the endoplasmic reticulum retention sequence and was constitutively located to the plasma membrane. Incorporation of E3gp19K-T1 in ORCA-010 resulted in permeabilization of the cell membrane of infected cells, in turn enhancing the release of new virions with good efficacy in prostate and ovarian xenografts in athymic mice. 

We previously generated an E1ACR2-deleted mutant that was designed to enhance potency in combination with apoptosis-inducing chemotherapeutic drugs by deleting the anti-apoptotic B-cell lymphoma-2 protein (Bcl-2) functional homologue E1B19K (Ad∆∆) [49]. In combination with cytotoxic drugs, Ad∆∆ showed superior efficacy compared to single drug regimens in preclinical models of drug-insensitive prostate and pancreatic cancers [50,51]. Synergistic tumour reduction was caused by improved viral infection followed by higher expression levels of the apoptosis-inducing and chemosensitising E1A-gene products that promote drug-induced DNA damage followed by aberrant mitosis and cell death [52]. Recently we further increased the selectivity and efficacy of Ad∆∆ by ablating Coxsackie and Adenovirus Receptor (CAR) binding and retargeting the fiber to αvβ6-integrins by inserting a 20 amino acid sequence from the Foot-and-Mouth-Disease Virus (A20FMDV) generating Ad5-3∆-A20T [53]. The αvβ6-integrins are frequently expressed in PDAC, breast and colorectal cancers but not in normal tissues [54,55]. Additional fiber-modifications improved specific uptake in tumour cells by reducing sequestration in the blood by preventing CAR mediated binding to erythrocytes and improved the liver-to-tumour viral ratios in murine models [56,57,58]. Furthermore, the *E3gp19K*-gene was deleted to promote MHC class I expression and reactivation of the host anti-tumour immune responses [53]. Ad5-3∆-A20T retained the potent synergy observed for Ad∆∆ with gemcitabine, and may be suitable for clinical translation targeting late stage PDAC lesions. The effectiveness and minimal toxicity to normal cells indicate that Ad5-3∆-A20T is a worthy candidate for combination therapies with cytotoxic drugs to safely and effectively kill treatment-resistant αvβ6-integrin expressing cancers.

A completely different strategy was employed when developing ColoAd1 (enadenotucirev, EnAd; PsiOxus Thrapeutics Ltd., Abingdon, UK), a potent chimeric Ad3/Ad11p recombinant selected by ‘directed evolution’ on the colon cancer cell line HT29 [25]. The highly efficacious recombinant ColoAd1 has the Ad11p backbone with the entire E3-region and a small E4-domain (E4orf4) deleted, in addition to a partial E2B substitution by the Ad3 E2B genes. Potency and selectivity was significantly higher compared to Onyx-015 and Ad5. The Ad11p capsid proved more resistant to elimination by human serum and blood cells than Ad5 and consequently, viral activity was better preserved after systemic delivery [59]. Interestingly, the mechanism for cancer-selectivity of ColoAd1 is not entirely understood although, the deregulated metabolic pathways in cancer cells have been indicated [60]. Infection with ColoAd1 resulted in a more rapid drop in ATP levels than with Ad5 and Ad11p, likely a consequence of the smaller viral genome and the shorter life-cycle of the recombinant. ColoAd1 lacks expression of E4orf4, which normally feeds back to limit E1A expression and regulates 5’ Adenosine Monophosphate-activated Protein Kinase (AMPK) activity that controls cellular energetics. The sudden fall in ATP levels resulted in membrane blistering and necrosis-like cell death with release of proinflammatory factors that stimulated T-cell activation and targeted tumour cells. An initial clinical trial with ColoAd1 investigating the mechanism of action demonstrated that both intratumoural and intravenous delivery was feasible [61]. Following this trial ColoAd1 entered phase I-II trials and promising outcomes in several solid cancers after systemic delivery were reported [60,61]. Currently a multicenter phase I trial is in progress including patients with metastatic or advanced epithelial cancers and a phase I trial targeting patients with recurrent platinum resistant ovarian cancers (NCT02636036; NCT02028117). 

Recently, a similar approach using a pool of viral serotypes including ColoAd1, resulted in the isolation of two recombinants that were selective for ovarian cancer cells [62]. Both recombinants retained the first 10–13 Kbp from ColoAd1 and the remainder of the genome was from Ad3. The findings from these studies and the trials with ColoAd1 suggest that species B viruses that bind to CD46 and/or desmoglein 2, both highly expressed on most tumour types, may have a potential advantage in future developments of oncolytic viruses. An important finding with ColoAd1 was the pro-inflammatory properties that indicate that type B adenoviruses may more efficiently stimulate long-term anti-tumour immunity than type C mutants [59,60]. 

In the second approach, tumour-selectivity is achieved by placing the viral genome under control of a tumour-specific promoter [26,63,64]. The E1A gene is the first to be expressed upon infection and has often been placed under control of tissue- or tumour-specific promoters [39]. For example, in the CG7870 virus, the E1A gene is under control of the prostate-specific rat probasin promoter and the E1B genes regulated by PSA promoter-enhancer elements, which are activated by the androgen receptor that is often deregulated in prostate cancer [65]. Another example is the Adv-TERTp-E1A mutant with E1A controlled by the human telomerase reverse transcriptase (hTERT) promoter that is frequently upregulated in cancer cells. Tumour selectivity was demonstrated in early phase clinical trials targeting solid cancers [27].

### Combination of Oncolytic Adenoviruses with Chemotherapy

Although the safety of replication-selective adenoviruses was proven in numerous clinical trials, single agent treatment with oncolytic adenoviruses have only resulted in moderate efficacy [26,66]. However, treatment efficacy was increased when the mutants were combined with chemotherapy [26,67]. 

The mitotic inhibitors paclitaxel and docetaxel prevent microtubule-depolarization, arresting the cell cycle and triggering apoptosis [68]. The combined treatment of paclitaxel and Onyx-015 resulted in synergistic effects in the treatment of non-small cell lung cancer (NSCLC) in vitro in cell lines and primary cultures obtained from lung cancer patients [69]. The combination of docetaxel or paclitaxel with CV787, in which E1A and E1B are placed under control of PSA and hKLK2 promoters, respectively, was reported to synergistically enhance cell death in LNCaP prostate cancer cells [70]. CV787-induced synergistic cell killing was suggested to be due to increased viral production and E1A-mediated sensitization to docetaxel.

Anti-metabolites such as gemcitabine, can alter nucleotide synthesis by targeting different key enzymes implicated in nucleotide production [26]. The combination of OBP-301 (Telomelysin^®^, E1A and E1B regions under the control of hTERT promoter) with gemcitabine (a nucleoside analogue chemotherapy) resulted in synergistic cell killing both in vitro and in vivo in H460, H322, H358 lung cancer cell lines [71]. The synergistic effect was caused by Telomelysin^®^-mediated S-phase entry, which was suggested to sensitise infected cells to gemcitabine. Telomelysin^®^ has completed a phase I trial confirming safety and activity in various solid tumours and, in addition, it is currently being tested in various Phase I/II clinical trials for hepatocellular carcinoma, esophageal cancer and melanoma patients (NCT02293850; NCT03213054; NCT03190824) [27].

Treatment with topoisomerase inhibitors induces double strand breaks, promoting cell cycle arrest in the G2-phase [72]. The combination of the AdΔ24 with the topoisomerase I inhibitor irinotecan resulted in enhanced anticancer effect both in vitro (in U-85 MG and U-25 MG human glioblastoma cell lines) and in vivo (intracranial xenograft models) [73]. This enhanced cell killing was suggested to be due to AdΔ24-mediated upregulation of topoisomerase I expression and activity and accumulation of cells in S-phase. Enhancement of cell death was also detected in esophageal carcinoma and in prostate carcinomas when Onyx-015 was combined with the topoisomerase II inhibitors Vp-16 (etoposide) or mitoxantrone [74,75].

We previously demonstrated that the mutant *dl*922-947 (E1ACR2- and E3B-deleted), synergistically enhanced mitoxantrone- and docetaxel-induced cell death in vitro (in PC3, DU145 and LNCaP prostate cancer cells) and in vivo in PC3 and DU145 xenografts [75]. The combination of *dl*922–947 with mitoxantrone or docetaxel resulted in increased viral uptake and E1A expression. To further enhance the cell killing efficacy and apoptosis induction of E1ACR2-deleted mutants, we developed the AdΔΔ oncolytic mutant (E1ACR2- and E1B19K-deleted) [49]. The E1B19K protein binds to pro-apoptotic Bcl-2 Associated protein X (BAX) preventing the formation of the Bcl-2 homlogous Antagonist/Killer (BAX-BAK) complex and the subsequent permeabilization of the mitochondrial membrane and apoptosis [35]. Intratumoural administration of the AdΔΔ oncolytic mutant caused synergistic cell killing and increased apoptotic death in combination with mitoxantrone or docetaxel in prostate cancer cells, and promoted tumour regression in murine PC3 and DU145 xenograft models [49]. The combination treatment of AdΔΔ and mitoxantrone was recently shown to promote increased apoptosis and attenuation of mitoxantrone-induced autophagy resulting in increased cell death in 22Rv1, PC3 and PC3-M prostate cancer cells [51].

The exact mechanisms for selective, synergistic cell killing in cancer cells are dependent on the specific genetic alterations in each cancer cell and oncolytic mutant [76]. However, increased drug-induced apoptosis in response to E1A expression has been indicated in combination with several cytotoxic drugs including inhibitors of metabolism, microtubules and topoisomerases. In prostate and pancreatic models, E1A-mediated caspase 8 and 3 activation through E1A-binding to the caspase 8 inhibitor cellular (Fas-Associated protein with Death Domain (FADD))-like IL(InterLeukin)-1β-Converting enzyme (FLICE) Inhibitory Protein (cFLIP) followed by mitochondrial depolarization significantly enhanced apoptosis in response to the cytotoxic drugs (e.g., Miranda et al., 2012) [77,78].

## 3. Oncolytic Immunotherapy

Recent developments in immunology including the clinical applications of immune-check point inhibitors, have greatly increased the understanding of oncolytic virus interactions with the host immune system. Accumulating evidence attribute the main efficacy of oncolytic viruses to the induction of tumour specific immune responses, initiated by viral lysis and tumour-antigen exposure. Viral genome amplification and replication cause tumour specific cell lysis, which releases tumour antigens, and pathogen- and damage-associated molecular pattern molecules that stimulate tumour-infiltrating antigen presenting cells that activate innate and adaptive immune responses [79,80]. Tumour cells often express higher levels of the immune checkpoint proteins (e.g., PD-1 ligands) and are therefore immunologically ‘cold’ [81,82,83]. A prevelant strategy is to utilise the lytic abilities of oncolytic viruses to alter the tumour microenvironment, turning immunologically ‘cold’ tumours ‘hot’. I Combination therapy with immune checkpoint blockade can then efficiently eliminate the tumours, as discussed in the reviews by Martin and Bell, and Breitbach et al. [84,85,86]. In addition, transgenes that promote local cytokine release and tumour infiltration of lymphocytes are often included in the oncolytic adenoviral genome such as, granulocyte macrophage colony stimulating factor (GM-CSF), interferon (IFN)-α, cluster of differentiation 40 ligand (CD40L), and interleukin (IL)-12 and -18 [87].

GM-CSF is an immune-modulatory cytokine that induces activation of monocytes and macrophages, and promotes T-cell-mediated systemic antitumor responses [88]. Re-activation of the host anti-tumour immune defence after infection with oncolytic adenoviruses expressing GM-CSF has been established in several preclinical models with numerous adenoviral mutants and in early phase clinical trials [44,89]. One of these mutants, replication-selective Ad5/3-∆24-GM-CSF, also known as Oncos-102, has been tested in patients with metastatic solid cancers resulting in stable disease or minimal responses in half of patients [90]. Results from a Phase I clinical trial with Oncos-102 in combination with cyclophosphamide demonstrated that the treatment was safe with no significant adverse effects while the best responses were stable disease in 40% of patients [81]. Further developments are required to realise long-term immunity and elimination of untreated lesions similar to that reported for the recently market-approved GM-CSF-expressing herpes virus T-VEC [91].

The cell surface receptor CD40 is expressed on all antigen presenting cells. The interaction of CD40 with its ligand CD40L (also called CD154) has been shown to prevent cell proliferation and promote apoptosis in malignant breast and ovarian carcinomas [92,93]. The CD40 activation plays important roles in humoral and cell-mediated immunity, enabling dendritic cells (DC) to mature and support activation of cytotoxic T-cells [94,95]. To take advantage of the overexpression of CD40 in the majority of breast cancer cells, the CD40L was expressed from the oncolytic mutant AdEHCD40L with replication controlled by a hypoxia-response element (HRE) and an estrogen response element (ERE) [96]. AdEHCD40L showed increased cytotoxicity, anti-tumour immune effects and apoptosis-induction in cancer cells only. Another CD40L-mutant, Ad5/3-hTERT-E1A-hCD40L (also named CGTG-401) with replication controlled by the hTERT promoter, selectively killed CD40-expressing cancer cells resulting in tumour regression and apoptosis in CD40-expressing xenograft models [97]. Safety of CGTG-401 and immunological responses have been proven in nine patients with solid tumours. Minimal responses or stable disease were reported in over half of patients [98]. Further developments by the same team generated the improved mutant LOAd703 [99]. In addition to improved and modified CD40L, LOAd703 also expresses the full-length human 4-1BB ligand (4-1BBL) to further stimulate innate and adaptive immune responses for efficient elimination of infected tumour cells. Safety and immunological responses are currently evaluated in Phase I/II trials in patients with metastatic pancreatic ductal adenocarcinomas in combination with gemcitabine or nab-paclitaxel (NCT02705196; NCT03225989). 

IL-12 is a proinflammatory cytokine with great potential to activate the host anti-tumour immune responses after oncolytic virus infection [100]. IL-12 activates both innate and adaptive immune systems by promoting antigen presentation and has been incorporated in many oncolytic adenoviruses. One example, is Ad5-yCD/mutTKSR39rep-hIL12 that expresses a mutant TK and IL-12 [101,102]. Infection with this mutant in combination with 5-fluorocytosine (5-FC) and valganciclovir in a prostate adenocarcinoma mouse model resulted in reduced number of metastasis and improved survival. Ad5-yCD/mutTKSR39rep-hIL12 is currently in Phase I clinical trials for patients with metastatic pancreatic cancer in combination with cytotoxic drugs and for locally recurrent prostate cancer after definitive radiotherapy (NCT03281382; NCT02555397). A concern with IL-12 therapy is the systemic toxicity of the cytokine. To address this issue, a modified non-secreted and consequently less toxic version of IL-12 was recently expressed from the highly efficacious Ad∆∆ mutant, previously developed in our laboratory, by replacing the E3gp19K gene (Ad-TD-nsIL-12) [103]. Deletion of the E3gp19K gene allows MHC-class 1 presentation of tumour antigens and is commonly included in oncolytic adenoviruses to facilitate reactivation of the host anti-tumour responses. In addition, the Ad-TD-nsIL-12 virus showed promising CD8+ T-cell dependent efficacy in immunocompetent Syrian Hamster models of pancreatic cancer. 

Taken together, immune stimulatory mutants, including those not mentioned here, show great promise for future clinical applications of oncolytic immunotherapy. Long-term improved tumour immunity is a probable outcome in combination with cytotoxic drugs, immune checkpoint inhibitors, cytokines and immune activators. 

## 4. Tropism Modification Strategies

### 4.1. Native Adenoviral Receptor Interactions

Adenoviruses have not evolved to be intrinsically tumour selective. Rather, in their wild state they cause transient and non-life threatening (at least in the immunocompetent host) infections of the respiratory and gastro-intestinal tracts, and ocular infections. This is due to the native Ad tropisms, which result in infection and spread of virus within healthy tissues. If left unrefined, Ad based virotherapies would have little uptake in cancerous cells, and exhibit reduced efficacy due to sequestration of the virus in non-target tissues, clearance of the virus prior to delivery to the target site, and consequent induction of dose limiting toxicities. Consequently, to achieve maximum therapeutic efficacy with minimal “off-target” toxicity, extensive refinement of these tropisms are required to tailor the virotherapeutic into a cancer selective agent.

Adenoviral serotypes bind different combinations of receptors, reflecting the diversity of natural adenoviral pathogenicity as reviewed by Ghebremedhin [104]. Of note is the fact that adenoviruses capable of binding the same receptors may do so with different affinities, exemplified by Ad11 and Ad21, where the latter has substantially lower affinity for, and a different binding mode to CD46 [105]. Receptor tropisms can be inferred from sequence alignments and predicted structural informatics, but often remain biologically unconfirmed. The modifications required for effective viral “detargeting” strategies depend upon the choice of adenoviral serotype used, and a detailed mechanistic understanding natural virus: host receptor interactions is therefore essential to enable rational modification of the capsid proteins. Previously characterized interactions, and mutations known to ablate them, are summarised in Table 1.

### 4.2. Ablation of Natural Adenoviral Tropisms

#### 4.2.1. CAR (Coxsackie and Adenovirus Receptor)

Perhaps the best described adenoviral tropism is CAR (Coxsackie and Adenovirus Receptor), broadly expressed in tight junctions of epithelial tissues [119,120]. Originally shown as the receptor for adenoviruses 2 and 5 [121], CAR has since been shown to be a receptor for a diverse range of adenoviruses across the species [122]. Crystal structures have been obtained for Ad12 [123] (species A) and Ad37 [124] (species D) in complex with the CAR-D1 domain; curiously the liganded structures have never been determined for the classical Ad5-CAR interaction or indeed any of the widely accepted Species C and B1 complexes. However, the Ad5-CAR interaction has been characterised in detail through mutational analysis of the Ad5 Fiber-knob domain [106,107,125,126]. 

**Figure 3:**
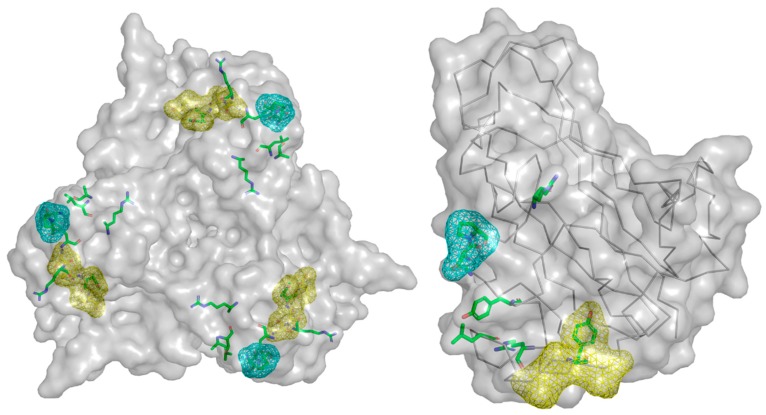
**Coxsackie and Adenovirus Receptor (CAR) interacting residues within the Ad5 fiber knob domain.** Known CAR interacting residues are shown as green sticks. The blue and yellow mesh shows the surface of the KO1 and ΔTAYT mutations, respectively. Structure from PDB: 1KNB.

Mutations to ablate the interaction between adenovirus and CAR have been well explored in the context of Ad5 [106,107,126]. The most efficacious, and thus most widely utilised, of these are the KO1 (the 1st of 10 mutations in the same study) and ΔTAYT mutants [106,107]. KO1, shown as cyan mesh in Figure 3, has been shown to almost entirely ablate the ability of Ad5 to bind CAR and to work in vivo, though it is not sufficient to alter the native viral hepatic tropism following intravascular administration [127,128].

The ΔTAYT mutation removes residues 489–492 in the FG loop of Ad5, shown in Figure 3 as yellow mesh. The TAYT motif was predicted to be important to receptor binding due to its high conservation between 14 adenoviral species (Ad2,4,5,8,9,12,15,17,19,28,31,37,40 long, and 41 long) [107], suggesting that this mutation may be effective in other Adenoviruses. The αvβ6 targeted Ad5-3Δ-A20T oncolytic virus shows the efficacy of this mutation in CAR binding ablation in vivo, though as with the KO1 mutation, ablation of CAR binding alone is insufficient to eliminate accumulation in the liver [53,129]. Though CAR-binding ablation may combat sequestration of adenovirus in the blood by preventing binding to CAR expressing erythrocytes, with the KO1 mutation being shown to prevent haemaglutination [56,130,131].

#### 4.2.2. CD46/MCP (Membrane Cofactor Protein)

CD46, also called Membrane Cofactor Protein (MCP), is a complement regulatory protein expressed on the surface of all nucleated cells except erythrocytes [132]. This near ubiquitous protein has been identified as important to the pathogenicity of several infectious agents. It protects against complement activity and has been implicated in the induction of regulatory T-cells, all covered in the review by Yamamoto et al. [133].

At time of writing, the only full length crystal structure of the CD46 extracellular domain was determined in complex with the Fiber-knob domain of adenovirus serotype 11 [134]. This is one of several Species B adenoviruses which have been shown to utilise CD46 as their primary cell entry receptor [105,135,136]. Crystallographic structures are available for Ad11 and Ad21 in complex with CD46. The authors of the Ad21 complex structure suggest that there may be two modalities of CD46 interaction governed by the length of the HI and DG loops [105,134,136].

**Figure 4:**
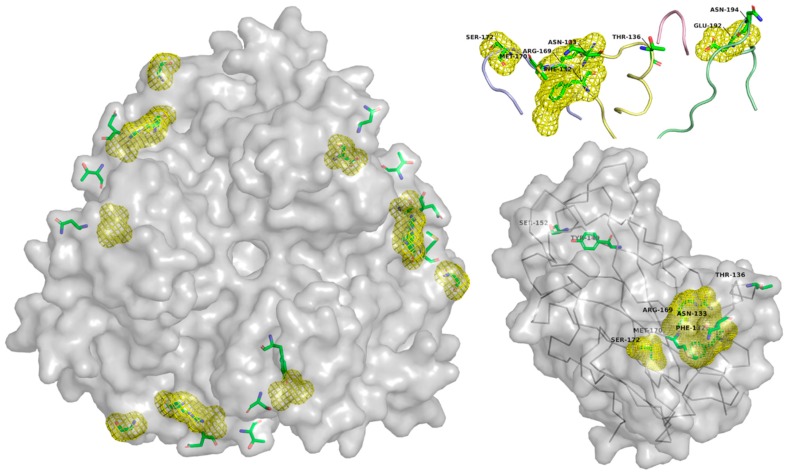
**Membrane cofactor (CD46) interacting residues, and known mutation sites, within the Ad35 fiber knob domain:** The key residues which interact with CD46 are shown as green sticks. The yellow surface shows the region in which known mutations which abrogate CD46 interaction occur. In the top right is a detailed view of the 4 loops which interact with CD46, HI (blue), DG (yellow), GH (red), and IJ (green). Structure from PDB: 2QLK.

Despite there being no liganded structure available for the Ad35 interface with CD46, the interaction is well explored, and the individual contact residues characterised, shown as green sticks in Figure 4 [108]. The analysis of CD46 contact residues reveals the locations of critical mutations which can be used to abrogate CD46 interaction, the locations of which are seen as yellow mesh in Figure 4 [108].

There is no published development of CD46 binding ablated vectors for therapeutic use. This is curious when compared to the widespread adoption of de-targeted CAR binding viruses. This may be a result of the perceived benefit of a universal entry pathway in favour of post infection regulation, despite the potential for immunosuppressive properties derived from activation of the CD46 pathway [137,138]. 

CD46 utilising adenoviruses remain a popular choice for the development of oncolytic virotherapies since CD46 is upregulated in numerous cancers, though CD46 is far from unique to cancer as it is present on the surface of all nucleated cells [139]. A good example is ColoAd1/enadenotucirev (EnAd), an Ad11p/Ad3 chimera, generated by recombination and selection of adenoviruses rather than rational modification, and has progressed to phase I/II clinical trials [25]. EnAd posesses promising oncolytic properties derived from several mutations, including E3 and E4 region deletions [25,140], and is purported to have a primary tropism to both CD46 and desmoglein 2, though the affinities have not been fully defined [59,141]. It has previously been suggested that the species D adenoviruses Ad26 and Ad48 may also utilise CD46; vectors derived from these viruses are currently under development as HIV and Ebola vaccines [142,143,144,145]. However this alleged tropism remains unclear, with other papers suggesting CAR as the primary receptor [146,147].

#### 4.2.3. Desmoglein 2

Desmoglein 2 (DSG2), part of the desmoglein subfamily of cadherin junctional adhesion protein and one of four DSG isoforms, is a cell entry receptor for species B adenoviruses 3, 7, 11, and 14 [148]. DSG2 requires knob domains to form a constellation to mediate cell entry as shown by studies of whole virus, dimerised knob domains, and penton dodecahedrons (a natural particle produced by Ad3 composed of 12 penton-fiber complexes which is capable of opening DSG2 mediated cell junctions) [149,150,151,152,153].

Like CD46 utilising viruses, efforts have focused on exploitation of this tropism rather than abolition of DSG2 binding. Work from the lab of David Curiel generated an Ad5 vector pseudotyped with the fiber-knob of Ad3 able to efficiently infect prostate cancer cells [154]. Further development led to the CGTG-102 virus [155]. CGTG-102, now branded as ONCOS-102, has progressed into phase 2 clinical trials in colorectal cancer and pleural mesothelioma having shown safety in a small phase I trial, while a similar virus showed efficacy in a phase I trial of ovarian cancer [81,155,156,157,158]. 

**Figure 5:**
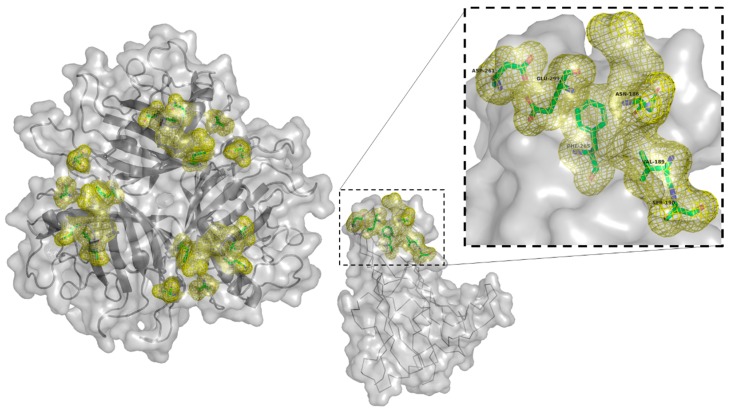
**DSG2 interacting residues, and known mutation sites, within the Ad3 fiber knob domain:** The key residues which interact with DSG2 are shown as green sticks. The yellow surface shows the region in which known mutations which abrogate DSG2 interaction occur. Structure from PDB: 1H7Z.

Work has been performed to increase the affinity of Ad3 fiber-knob for DSG2 in pursuit of a therapeutic molecule capable of loosening epithelial cell junctions to improve tumour penetration culminating in the JO-4 molecule [151,159,160,161]. Whilst in search of enhancing mutations the lab of Lieber identified several mutations capable of reducing or ablating the Ad3-DSG2 interaction, the locations of which are seen as yellow mesh in Figure 5. When mapped to the Ad3 fiber-knob crystal structure all of these mutations are at the extreme distal end suggesting the location of the binding interface which may overlap with that of GD1a glycan [109,162].

It has been suggested that adenovirus binding DSG2 can induce an epithelia to mesencymal transition (EMT)-like phenotype, and induce transient opening of cell junctions [148,163]. Presumably this facilitates viral spread through the tissue. However, EMT has also been linked to cancer metastasis, though it is unclear if EMT is strictly necessary for metastasis and remains an area of intense investigation [164,165,166,167]. DSG2 interacting molecules may be capable of inducing EMT/MET mesencymal epithelial transition (MET) and treatments based on this ability must be monitored for this phenomenon.

#### 4.2.4. GD1a Glycan and Sialic Acid

Unlike the receptors discussed so far GD1a is not a protein, but a large sialylated polysaccharide attached to various glycoproteins. Sialylated glycans are expressed on the surface of numerous glycans and is the receptor for many viruses [168]. Interestingly, Ad37 has been shown to specifically bind and utilise the GD1a glycan for cell entry, rather than any sialylated glycoside. The asymmetric interaction with the fiber-knob means only one copy of the GD1a can interact per fiber-knob domain, in contrast with the three copies of CAR and CD46 [110].

GD1a glycan has been shown to act as an entry receptor for numerous species D adenoviruses. In addition to the conservation of several key GD1a binding residues, preincubation of Ad3, 8, 9, 19p, or 37/19a fiber-knob protein with soluble GD1a inhibited their binding to human corneal cells to varying degrees [110]. This further corroborates other reports that Ad3, 19p, 37, and 52 complex with sialic acid [169,170,171]. Interestingly adenovirus 52 and 37 have both been modelled in complex with CAR and sialic acid simultaneously, demonstrating the ability of adenoviruses to bind two different classes of receptor at once [130,171].

**Figure 6:**
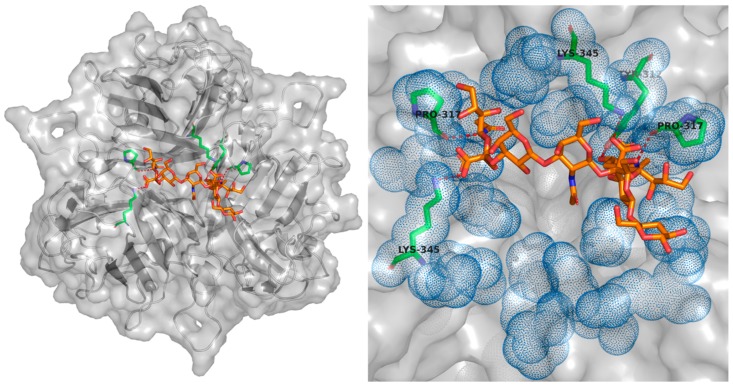
**GD1a/Sialic Acid interacting residues within the Ad37 fiber knob domain.** Key residues forming the GD1a-Ad37Fkn interaction are shown as green sticks, with the GD1a in orange, hydrogen bonds are shown by red dashes. While the interface can occur in three orientations, only one set of interacting residues is shown. The blue dots show the surface of all residues shown to be able to interact with GD1a or support the interaction, seen to create a large apical binding pocket. Structure from PDB: 3N0I.

No mutations to ablate Sialic acid/GD1a binding have yet been reported in adenoviruses. Nevertheless, Tyr312 and Lys345 residues are shown to be critical for GD1a binding in Ad37 and are conserved in other species D adenoviruses [110]. These and the supporting Tyr308, Pro317, and Val322 residues present obvious mutational targets but remain untested. These residues are shown in Figure 6 as green sticks.

A recent study from the Arnberg lab reveals that Ad52, a species G adenovirus with two different fiber proteins (SFK—Short Fiber, LFK—Long Fiber), binds to polySia (α-2,8-linked poly sialic acid) via its SFK [172]. This study demonstrates that Ad37 fiber-knob was capable of polySia interaction whilst Ad52SFK interacts with other sialylated glycans, but at a markedly lower affinity. The authors demonstrate a primary hydrogen-bond interface with the first sialic acid of the polySia and use molecular dynamics simulations to argue convincingly for transient electrostatic contacts, which further stabilise the interface, explaining the increased affinity with polySia chains ≥3 units.

PolySia is an unusual post-translation modification (PTM) with expression largely restricted to neurological tissues of the hippocampus, olfactory bulb, and hypothalamus in healthy adult brains [173,174,175]. Though it has been suggested that polysialylated protein being involved in the development of other organs via polysialylated Neuronal Cell Adhesion Molecule (NCAM) and in innate immunity via polysialylated CCR7; these are two of just nine reported carrier proteins [176,177].

While a neurological tropism is of potential concern when engineering a virus for cancer virotherapy, it is worth noting that several cancers including glioma, astrocytoma, neuroblastoma, and Non-Small Cell Lung Cancer (NSCLC) over-express polySia [178,179,180,181,182,183,184]. While usage within the brain seems likely to generate off target effects within healthy polySia expressing tissues, however, this tropism could have potential for usage in vectors for NSCLC or neuroendocrine tumours when used in conjunction with the appropriate selectivity mutations.

These studies suggest several key binding motifs, but also a degree of flexibility in the mode of interaction with sialylated glycans. It should be possible to ablate the primary interface by mutation of the key protein-carbohydrate bonds forming residues. However, the electrostatic interface may retain some limited ability to interface based on the Fiber-knobs surface charge. This seems unlikely to impact biodistribution in the presence of a strong retargeting receptor, though this electrostatic interaction in the knob could potentially be defeated by careful selection of charged residues for mutation.

#### 4.2.5. Blood Coagulation Factor X (FX) and Heparan Sulphate Proteoglycans (HSPG)

HSPG’s are expressed ubiquitously on cell surfaces and represent the receptor for myriad viruses including all viruses with well described oncogenic properties (except Epstein-Barr virus) [185,186]. Adenoviruses have been shown to interact with HSPG’s through differential mechanisms. Ad2, 3, and 5 have all been shown to interact with HSPG’s in a sulphation dependent manner (higher sulphation increases affinity). The interaction is mediated by a BBXB motif (B is a basic residue, X is a hydropathic residue) typified by the KKTK motif in the Ad5 and Ad2 Fiber-shaft, one of two consensus sequences proposed for heparan sulphate glucosaminoglycans (HSGAG), the other being BBBXXB [187,188,189,190].

The suggestion that the KKTK motif and subsequent HSPG interaction was solely responsible for trafficking of adenovirus to the liver has been largely dispelled by subsequent studies utilising mutantions or deletions in the KKTK motif. Chimerisation with the fiber proteins of Ad31 and Ad41 (CAR-interacting adenoviruses lacking the KKTK motif), the S* modification (Ad5 residues _91_KKTK_94_ → _91_GAGA_94_), and the _91_KKTK_94_ → _91_RGDK_94_ mutation to substitute KKTK for an additional integrin interacting motif, all abolish HSPG binding, but leave the hepatic tropism intact [114,115,191]. These studies also demonstrated an important role for the fiber shaft KKTK motif in making the fiber shaft flexible enough to be able to bend and allow subsequent engagement between the viral penton base protein and cellular αvβ3/5 integrins. Mutation of these key residues (as in the S* mutation) render the virus fiber shaft inflexible and thus the virus fitness is severely compromised.

More recently, it has been demonstrated that adenovirus liver transduction is actually mediated by a high affinity, Ca^2+^ dependent “bridging” interaction between the major capsid protein, hexon, and blood coagulation factor X (FX) [192]. CryoEM was able to identify the hexon hypervariable regions (HVR) 3, 5, and 7 as critical interaction determinants [57,111,112]. Mutation of HVR5 and 7 has been shown to efficiently abrogate Ad5 trafficking to the liver by ablation of the Hexon: FX interaction through both rational modification, and pseudotyping of the Ad5 HVRs with the HVRs of non-FX interacting adenoviruses, namely Ad26 and Ad48 [111,113,193,194]. The HVR7 mutant in particular was effective in detargeting Ad5 from the liver in vivo [111].

Coagulation factor X has been shown to shield Ad5 from complement mediated attack, via inhibition of IgM binding [195]. Consequently, it is conceivable that ablation of this interaction may result in enhanced clearance of the FX binding ablated virus from the body, reducing therapeutic efficacy [196]. However, mutation of hexon protein to ablate FX interactions could be extended to also inhibit IgM binding, albeit, this may preclude the potentially beneficial effects provided through steric inhibition of hexon interaction by large molecule binding (such as FX), which may represent a broadly effective mechanism for inhibiting immune recognition, as described by Schmid et al. (discussed later in this review) [12]. Not all adenoviruses bind FX, highlighting that binteraction with FX is not a universally required mechanism for immune evasion, and this immune evasion could be achieved through incorporation of a hexon or hexon hypervariable regions derived from low seroprevalence adenovirus that are unrecognized by the immune system [197]. Therefore, whilst ablation of FX binding may have effects on adenovirus clearance the virus must be considered individually in the context of its native FX binding ability and seroprevelance in the population.

Uptake into to the liver hepatocytes occurs via a secondary interaction of the Ad5:FX complex with cellular HSPGs forming an adenovirus: FX: HSPG complex which is then internalised by the cell [198]. This uptake pathway was subsequently found to be particularly dependent on O-linked sulphate groups, which are abundant on hepatocytes [199]. The fiber protein continues to exert influence over the final tropism, with Ad5/35Fkn pseudotyped viruses exhibiting differential liver tropism to Ad5 when ablated for FX interaction [200]. While the mechanism underlying this effect remains undetermined, pH dependent effects on endosomal transport may relate to differential properties of the Ad35 vs. Ad5 fiber proteins in endosomal escape, rather than a direct interaction with HSPG.

While most development has focused on ablation of HSPG binding, this interaction has been utilised in therapeutic development. The Ad5.pk7-Δ24 virus which contains a poly-lysine motif in the in the C-terminus of the fiber protein to facilitate CAR independent infection via HSPG [201].

These studies highlight that FX interaction must be eliminated to effectively disrupt HSPG interaction. However, the effectiveness of the poly-lysine modification, and the ability of peptides to form electrostatic interactions implies that absolute abolition of HSPG binding may be unfeasible given the charged adenovirus capsid [201,202]. HSPG’s electrostatic mode of interaction means any patch of positive charge on the adenoviral capsid represents a potential binding site. Given the variability of adenoviruses net electrostatic potential it seems likely that the robustness of the aforementioned mutations will vary depending upon serotype [203]. However, if interactions are brought to sufficiently low affinity this is unlikely to have in vivo relevance.

#### 4.2.6. Integrins, ανβ3/5

The integrins ανβ3 and ανβ5 were shown to be required for internalisation of Ad2 in 1993 via interaction with the penton base protein where it becomes internalised through clathrin coated pits [204,205]. This endocytosis is mediated by phosphotidylinositol-3-OH (PI3K)-dependent Rho GTPase reorganisation of the actin cystoskeleton [206,207]. Integrins interact via a canonical RGD motif which is widely conserved despite occurring within a hypervariable region of the penton base protein [116]. Mutation of the RGD motif to RGE is sufficient to prevent efficient infection; RGE mutant Ad2 virus is shown to accumulate on the cell surface and not internalise and the mutation has been shown to be effective in an Ad5 background [116,126,204,208]. Alternatively, there is the EGD mutation which achieves similar ends [117]. This integrin mediated endocytosis mechanism has been shown to apply across almost the whole adenoviral species, with only the Ad40 species F serotype reported to have a naturally occurring RGA rather than an RGD motif [116,209].

There are reports that ανβ1, αMβ2, αLβ2, and α_IIb_β2 may also enable adenovirus internalisation in the absence of ανβ3 and ανβ5, though these interactions are less well described [210,211,212,213,214]. The RGD interacts with an NPXY motif, conserved at the C-terminus of integrin β-subunits; this motif is sometimes extended to include an N-terminal phenylalanine (FNPXY) [215,216]. Alteration of this motif has been shown to limit adenoviral cell penetration [217]. Given the diversity of α-subunits in the integrins, which adenovirus has been observed to complex and the conservation of the C-terminal NPXY motif in the β-subunits, it seems plausible that adenoviruses only dependency in terms of penton: integrin interaction is the presence of a β-subunit.

The complex between Ad2 and Ad12 with ανβ5 has been solved by CryoEM [218]. This study shows that integrins bind the penton at 5 RDG sites and form a continuous ring structure with ~60 Å spacing between the 5 RGD sites (similar to that of Foot and Mouth Disease Virus—FMDV) [219,220]. The authors suggest that this clustering of integrins is what stimulates endocytosis. An updated CryoEM structure of Ad12 in complex with ανβ5 reveals that steric hindrance limits integrin engagement to four copies per penton, despite pentavalency [214]. Comparison of the Ad2 and Ad12 complexed structures reveal that the more stable conformation of RGD in Ad12, constrained by lower flexibility of the loop on which RGD resides, enables more stable integrin interaction presenting an opportunity to engineer tighter integrin interaction and potentially modulate the viral dependency upon fiber mediated interactions [218].

Whilst normally acting as a co-receptor, it has been reported that ανβ5 binding alone is capable of facilitating efficient Ad5 infection and can interact with the penton with picomolar affinity (K_D_ of 1.4 × 10^-10^ M) as determined by Ad5 virus binding to CAR^low^ MDA-MB-435 cells [221]. It is worth noting that this study does not preclude non-intregin non-CAR means of cell uptake and would benefit from being revisited with surface plasmon resonance experiments to determine virus-integrin affinities in isolation. It was previously predicted that engagement of ανβ5 was not required for viral attachment, and while this may be true when cells bear a high affinity primary receptor the aforementioned study suggests it can represent a viable means of cellular attachment [204].

It has previously been suggested that insufficient flexibility of the fiber-shaft would preclude efficient infection due to an inability to bend to enable integrin interaction after primary receptor interaction [222]. Recent CryoEM of a mutant Ad5 virus with the short fiber protein of Ad35 (Ad5F35) indicates that their prediction that short shafted viruses will have limited flexibility is correct. However, it does not appear to preclude binding between the penton and integrin, and shows simultaneous interaction between short-fibered adenovirus with their primary receptor and integrins is possible [223]. Thus, when engineering adenovirus it is insufficient to rely upon the primary tropism to retarget the vector, and the penton RGD motif must be ablated to prevent off target infection.

#### 4.2.7. Scavenger Receptor (SR-A6)/Macrophage Receptor with Collagenous Structure (MARCO)

In vivo mouse studies show that macrophage depleted mice have greatly reduced inflammatory response to adenovirus infection [224,225]. This effect was shown to be due to reduced adenovirus infected MARCO^+^ macrophages local to the splenic marginal zone [226]. It is now clear that MARCO has a direct role in Adenovirus recognition and is needed for anti-adenovirus cytokine response, via the cGAS/STING pathway [227]. This effect has now been observed in species B (Ad35), C (Ad5), and D (Ad26) adenoviruses, and deletion experiments in the hexon imply that the site of interaction involves Hyper Variable Region 1 (HVR-1) [118]. However, the exact nature of the MARCO: hexon interaction remains uncharacterised. With cGAS/STING being identified as a key sensor of adenoviral infection, and innate antiviral/DNA sensing pathways becoming increasingly important to the field of oncolytic virology in general, further studies of this interaction are paramount [228,229,230,231].

## 5. Retargeting of Adenovirus by Engineered Receptor Tropism

Once natural tropisms have been ablated, in order to generate a cancer targeted virotherapy, it is necessary to provide an alternative means of infecting the cell; one which is specific to the tissue of interest. The choice of new cell entry receptor is limited by several factors: it must be expressed on the cell surface, selectively expressed/upregulated in the target cells, capable of internalisation following receptor binding and capable of being targeted by genetic modification of adenoviral capsid proteins.

The pool of cancer specific ligands is not so large as to make this a trivial feat. Accordingly, many studies have attempted to engineer a universal vector platform by utilising established ligand specific molecules, detailed in this section. Others have sought to utilise naturally occurring tropisms from other sources, but both methodologies pose challenges. An important benefit of the chimeric retargeting approach is highlighted in a study of a “knobless” adenovirus in which the fiber protein was engineered to have 7 shaft repeats, a trimerization motif, and the anti-Taq polymerase Z_taq_ affibody; this generated the Ad5/R7-Z_taq_-Z_taq_ virus based on Ad5 [232]. This proof of concept study showed a decrease in neutralisation by sera containing nAbs (neutralising antibodies) against adenovirus, which is hardly surprising given the deletion of most of a major viral capsid protein. However, the ability of these viruses to evade neutralisation in some sera highlights the important observation that some patients appear to neutralise adenovirus solely via the fiber-knob protein rather than the hexon, as is often cited [233,234].

### 5.1. Chimeric Fusion Proteins

#### 5.1.1. Single Chain Antibodies

By far the most prolific example of a protein with targetable affinity is the antibody, so it is logical that many have proposed the use of single chain variable antibody fragments (scFv’s) incorporated into the viral capsid. However, most scFv’s require the formation of disulphide bonds, formed in the endoplasmic reticulum [235]. Adenovirus proteins fold in the reducing environment of the cytosol, followed by packaging in the nucleus, creating a fundamental biosynthetic incompatibility limiting the effectiveness of this approach [236].

One example of a successful scFv fusion protein is the Ad5FFscFv47-CMV-GFP virus. An scFv fragment of an anti-IL13Rα2 (a selective marker of glioma) mAb was fused in place of the fiber-knob with a T4-fibritin trimerization motif between the scFv and shaft [237]. Whilst the resultant virus selectively infected glioma cells based on IL13Rα2 expression, the production process demonstrates the difficulty obtaining such a stable scFv. Selection of a viable scFv from the parental hybridoma required extensive biopanning. An attempt to solve this was by retargeting a pIX-scFv fusion protein with an ER trafficking signal [238]. Whilst this solved the folding issue, the pIX-scFv fusion integrated into the virus inefficiently. The authors addressed this problem by using an sdAb (single domain antibody). Though efficacious, this also requires extensive re-engineering of the targeting ligand, nullifying the primary advantage of scFv use: the wide availability of ligand specific reagents.

**Figure 7:**
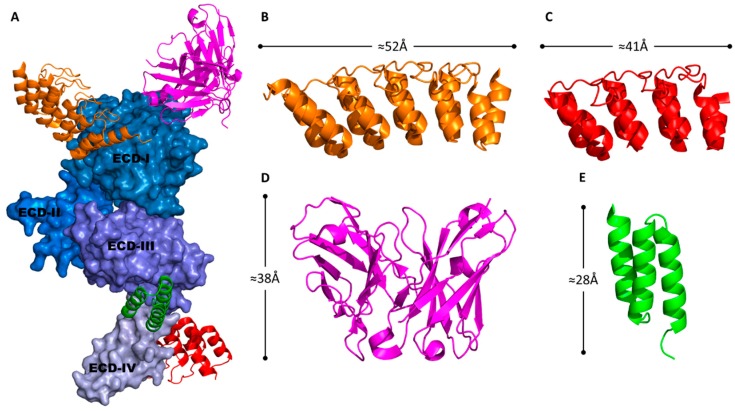
**Overview of antibody-like proteins specific to Human Epidermal growth factor Receptor 2 (HER2) which have been genetically integrated into Adenovirus.** HER2 is composed of 4 domains, Extra Cellular Domain (ECD) I-IV which can be bound by different proteins (**A**). The Designed Ankyrin Repeat Proteins (DARPIN’s) 9.29 (**B**) and G3 (**C**) are seen in orange and red complexing ECD-I and ECD-IV, respectively. ScFv chA21 (**D**) is seen in purple complexing ECD-1 (this particular ScFv has not previously been integrated to Adenovirus), and affibody ZHER2 (**E**) is seen in green binding at the ECD-III/IV interface. All molecules are shown to scale, structures from PDB: 4HRL, 4HRN, 3H3B, and 3MZW.

The proposed solution has been scFv’s forming a tertiary complex, altering the scFv sequence to contain a virus specific domain as well as its ligand specificity. An example is the addition of dimeric leucine zippers, where the scFv (still expressed from the viral genome) contains one half of the pair with the other expressed in place of the fiber-knob C-terminal of a trimerization motif to retain stability [239]. A similar approach utilised a bispecific diabody, a genetic fusion of two scFv’s with different specificities. This molecule had specificity for CD105 (endoglin) for targeting of vascular tissues and adenovirus fiber-knob [240]. Another utilised a fusion of Ad5’s native CAR receptor with an anti-HER2 scFv [241]. A similar scFv (chA21) is seen in Figure 7A,D, though this particular version has not been integrated into adenovirus.

However, at this time, no adenovirus targeted by means of a bridging molecule has been translated to the clinic. The dependence of these vectors on a non-covalent molecular interaction for retargeting demands high affinity binding to prevent off target effects with uncomplexed virus. A potential solution to this is expression of the adaptor from a virus which is ablated for all-natural tropism. However, this is yet to be attempted in the context of an adaptor:virus targeting complex and presents manufacturing issues. More problematic is providing evidence that the adaptor molecule itself does not have detrimental interactions. These issues create severe regulatory hurdles, leading many to abandon adaptor molecules as a means of retargeting and turn to more stable antibody mimetic proteins, which can be integrated into the viral capsid directly.

#### 5.1.2. Affibodies—FGFR2

Affibodies are antibody mimetic proteins based on a stable scaffold, in this case a 3-helix bundle derived from *Staphylococcus* bacteria Protein A. They are popular and versatile owing to their small size (~6.5 kDa), the ability to achieve picomolar affinities, and ability to correctly fold in the cytosol [242,243].

One of the earliest attempts to retarget adenovirus with an affibody was a proof of concept study where an anti-antibody Fc domain affibody was fused to the fiber protein, replacing the knob domain [244]. The authors demonstrated that the modified virus can specifically infect modified 293 cells displaying the Fc on their cell surface, but not wild type (WT) cells. However, the retargeted virus has a lower infectivity than the unmodified Ad5.

This strategy was then used to target cancer cells via an antibody against the well-known cancer marker HER2/neu (AKA ERBB2) [245]. The FibΔCAR-HI-Link-ZHZH fiber chimera containing 22 shaft repeats, an ΔLT_485,486_ deletion mutation to ablate CAR affinity, and a head to tail dimer of the anti-HER2 affibody “ZH” in the knob HI loop (seen in Figure 7A,E), was integrated into the Ad5/EGD vector (EGD being an integrin binding ablation mutation described in a previous study of affibody candidate molecules) [117]. The Ad5/EGD/FibΔCAR-HI-Link-ZHZH virus was able to infect HER2 expressing SKBR-3 (breast carcinoma) and SKOV-3 (ovarian carcinoma) cells with greater efficiency than WT Ad5 or non-integrin ablated chimeric vectors demonstrating the effectiveness of this approach. A comparable study using alternative anti-HER2 affibodies designed with an N-terminal fold on trimerization motif (derived from T4 fibritin) entirely replacing the knob domain showed similarly effective results [246].

The Ad5/EGD/FibΔCAR-HI-Link-ZHZH was further developed to include an _91_KKTK_94_ → _91_RKSK_94_ mutation in the fiber-shaft, and renamed Ad-ZH/3 [247]. When tested in mice bearing HER2^high^ PC346C prostate cancer tumours the Ad-ZH/3 cohort had significantly prolonged survival compared to mock. However, survival was not significantly improved vs. Ad5WT treated mice. Analysis of tumours from mice revealed that tumours from the Ad5WT treated mice retained HER2 expression in >40% of tumour mass, while Ad-ZH/3 treated tumours were negative for HER2. As the authors note, this suggests that the virus has effectively infected and killed HER2 positive tumours, but left the remainder of the tumour mass to grow unchecked.

This outcome is unlikely to be so clear cut in an immune competent model given the immune stimulatory effects (now accepted as a primary mode of action for oncolytic viruses) are likely to activate a T-cell responses against neighbouring cancer cells [248,249,250,251,252]. Yet it is a reminder of the danger of monotargeted therapies, something which has been considered in proof of concept experiments using two affibodies, with different targets, inserted into the Fiber HI-loop to create a virus with dual specificity [253].

The most recent example of a virus retargeted using an affibody is adenovirus serotype 43 virus pseudotyped with a similar affibody-knob chimera, also against HER2. The use of adenovirus Ad43 leverages the low levels of pre-existing immunity to the rare species D adenovirus and lack of cross neutralisation by anti-Ad5 nAbs [254]. Whilst the virus can efficiently transduce HER2^+^ cells, it is hampered by poor production titres due to inefficient incorporation of the chimeric fiber.

An intriguing departure from fiber-chimeras is the integration of an anti-HER2 retargeting affibody to the pIX protein, C-terminal of an engineered cathepsin cleavage site [255]. Integration of peptides at the pIX C-terminal has been shown not to interfere with viral assembly [256,257,258,259]. However pIX activity is required for dissociation of the fiber from the capsid during endosomal escape, to facilitate efficient infection [260]. So the authors foresaw, in line with earlier predictions, that high affinity association between pIX fusion proteins and ligand may prevent endosomal escape [261]. cathepsin is naturally present inside endosomes [262]. The authors inclusion of a cathepsin cleavage site N-terminal of the affibody enabled transduction of SKOV-3 spheroid cultures with efficiency greater than both Ad5WT or the pIX fusion lacking the cathepsin site, presumably by enabling cleavage of the affibody post-endocytosis and thus dissociation from the endosome.

Overall affibodies represent an attractive means of adenoviral retargeting, however the chimeric fibers are often to blame for poor production titres, a limitation likely to severely hamper their potential for clinical translation. It is worth noting that none of the affibody retargeted viruses have included Factor X binding ablation mutations, which can result in sequestration of the virus in the liver. The extent of off target effects in the liver cannot be determined due to the lack of in vivo imaging of the infected mice, except for the Ad43 vector, which is shown not to interact with FX naturally. Further development of these viruses must address these concerns.

#### 5.1.3. DARPins

Another class of antibody mimetic molecules are Designed Ankyrin Repeat Proteins (DARPins). Similarly to affibodies (and many modern antibodies) they are generated by library generation from a stable scaffold (in this case ankyrin proteins) and biopanning [263,264]. They differ to affibodies with a larger MW (~14–28 kDa) and are structurally distinct, but are otherwise similar in terms of the engineering opportunities in the context of adenovirus [265].

Despite the free N and C-termini of DARPins, there has been little development of them as adenoviral fusion proteins. While similar adaptor strategies have been employed as with affibodies, enabling retargeting to H-Ras and HER2 (seen in Figure 7A–C), the DARPins are expressed from *E.coli* and conjugated to the adenovirus prior to application [266,267,268]. The lack of genetic incorporation technically places the development of DARPins as adenoviral adaptor molecules beyond the scope of this review, nevertheless, given the clear opportunity to incorporate these robust and widely engineered molecules into the capsid it is pertinent to include them.

A dimeric DARPin molecule, joined by a linker, was generated with specificity to HER2 and adenovirus knob domain with an N-terminal SHP trimerization motif. This molecule has previously been reported to enable HER-2 retargeting of Ad5 [268]. The authors used this in concert with a novel antibody derived construct, an ScFv (mAb 9C12) against Ad5 hexon trimerized with an N-terminal SHP motif. The authors demonstrated that the trimerized ScFv was capable of pico-molar binding affinity for Ad5 (K_D_ 10.4 pM). When complexed to the Ad5 virus the DARPin construct efficiently retargeted the virus to HER2^+^ tumour cells, while the ScFv shielded the virus from neutralising antibody activity [12].

This impressive feat of protein engineering presents a clear opportunity to synthesise the DARPin retargeting molecule *in cis* with the virus, either as a transgene or fusion protein with the fiber or pIX proteins. Similarly, the efficacy of the shield adaptor may well be achieved by modification of the hexon hypervariable loops to eliminate immunogenic epitopes.

#### 5.1.4. scTCR Chimeric Fiber Proteins

An innovative study by Sebestyen et al. used a chimeric fiber protein expressing a single chain (sc) T-Cell Receptor (TCR) specific to Melanoma Associated antigen-A1 (MAGE-A1): Ad5.R1-scTCR as a targeting protein, rather than an antibody mimetic molecule [269]. The fiber consisted of the N-terminal region of the Ad5 fiber-shaft, the first pseudo-repeat of the shaft, followed by the Neck Region Peptide (NRP) trimerization motif and a single chain TCR specific to Human Leukocyte Antigen-A1 (HLA-A1) presented MAGE-A1 antigen. The virus was able to initiate productive infection in an epitope specific manner, displaying efficient infection and transgene expression in cells expressing a MAGE-A1 epitope, but not MAGE-A2 or MAGE-A1 negative cell lines.

The melanoma associated cancer-testis antigen MAGE-A1 is one of numerous MAGE cancer associated antigens. Their upregulation on cancer cells when normally restricted to germ cells makes them an attractive target for therapeutic development owing to the potential for cancer selectivity [270,271].

While an approach dependent upon antigen recognition by TCRs is inherently restricted, both by patient HLA type and the availability of high affinity scTCRs, this presents an effective method of targeting cancer neo-antigens with virotherapy. As such it is intriguing that this is the first and last reported usage of a TCR retargeted adenovirus, such a vector could surely benefit from modern capsid detargeting mutations. Especially in light of more recent work demonstrating the potential effectiveness of oncolytic adenoviruses targeted to MAGE-A1, utilising non-genetic targeting technique (electrostatic coating of Ad5 based conditionally replicating vector with MAGE-A1 peptide), and the ongoing development of therapeutic TCR molecules [202,269,272].

### 5.2. Peptide Based Retargeting of Adenovirus

There have been many attempts to retarget adenovirus by integration of peptides specific to ligands of interest. It has been shown that peptide insertion into the fiber-knob can exceed 100 residues, 50% larger than the knob itself, without major detrimental activity [273]. Though this is likely highly dependent upon the character of the inserted sequence.

Approaches attempted so far include incorporation of peptides into the adenoviral fiber-knob to target Epidermal Growth Factor Receptor (EGFR) or Fibroblast Growth Factor Receptor 1 (FGFR1) [274]. “Deknobbing” of the virus by removal of the Fiber-knob domain, and replacement with a trimerization motif and integrin binding RGD ligand [275]. Directed evolution approaches displaying peptides on the C-terminal of the fiber-knob to generate affinity to a glioma, pancreatic cancer, Transferrin receptor, and thyroid carcinoma [276,277,278,279]. Further specific examples of peptide incorporation and a discussion of non-fiber-knob incorporation sites including the pIX C-terminus are given in the excellent review by Dmitriev et al. [280].

#### Targeting αvβ6

ανβ6 Integrin is an oncofoetal antigen, highly expressed in many aggressively transformed and invasive cancers but absent in healthy tissues making it an ideal target for virotherapy [281,282]. FMDV naturally uses ανβ6 as a receptor, attaching to the integrin via a relatively simple interface in a semi-helical loop [283,284]. It is from this interface that the A20FMDV2 peptide (A20) was derived, a 20-mer with the amino acid sequence NAVPNLRGDLQVLAQKVART, and shown to specifically interact with ανβ6 at high affinity [285,286]. A20 was integrated into the HI loop of Ad5 Fiber-protein by genetic modification and was shown to enable efficient infection of ανβ6 positive cells in a CAR independent manner [287].

Integration of the TAYT in order to ablate the native Ad5 CAR tropism, which is not abolished by the integration of the A20 peptide, reduced hepatocyte tropism and uptake by liver resident macrophages (Kupffer cells) [58]. However, this was not sufficient to enable the virus to permeate the tumour mass in tested mice. The A20 peptide was incorporated into the Fiber-knob loops (CD and IJ) of the rare species D adenoviral serotype Ad48, replicating the success of the Ad5.A20 vector. Ad48, in contrast to Ad5, was shown to be insensitive to neutralisation by serum, raising interest in improved systemic delivery [288].

Several Ad5 based viruses were developed with and without the Ad48 Fiber-knob pseudotype, or the CAR binding ablation (KO1) mutation: Ad5.HI.A20, Ad5.KO1.HI.A20, Ad5/48kn.DG.A20. Respectively, these viruses were shown to transduce primary epithelial ovarian cancer (EOC) cells with 70, 60, and 16-fold improvements in affinity compared to Ad5, and 160, 270, and 180-fold improved affinity in αvβ6^high^/CAR^low^ BT-20 triple negative breast cancer cells. Importantly, these modified A20 vectors appeared capable of infecting EOC cells in the presence of highly neutralising anti-Ad5 neutralising immunity, demonstrating nearly 1000 fold improvement in the transduction of EOC cells following pre-incubation with adenovirus neutralising ovarian ascites [128].

The most recently published successful implementation of the A20 peptide retargeting technique is in the Ad5-3Δ-A20T virus in pancreatic cancer models. In this virus several early phase viral genes were also deleted (3Δ): E3gp19K to promote antigen presentation, E1ACR2 to render the virus conditionally replicative in tumour cells, and E1B19K to prevent inhibition of apoptosis [49]. The A20 FMDV2 peptide was inserted into the Ad5 fiber-knob protein, which was further modified with the TAYT CAR ablation mutation (A20T). This represents one of the most exquisitely engineered adenoviruses described to date, combining manipulation of the viral replication cycle via manipulation of the early genes to enhance cancer specific viral replication, with capsid modifications to improve viral specificity to tumour targets. The Ad5-3Δ-A20T was shown to kill effectively in cocultured pancreatic cancer and stromal cells, and prolong survival in xenograft models of pancreatic cancer in mice [53].

Despite this success, peptide retargeting is still limited by two major factors, which remain unaddressed in the field of peptide retargeted viruses: the availability of high affinity peptides for relevant cell markers and the inability to post-translationally modify adenoviral proteins. While modern biopanning techniques can raise many suitable peptides they are constrained by their nature as (usually) short 1-dimensional molecules, a structural constraint which frequently fails to translate into meaningful retargeting in the context of a 3-dimensional viral protein.

This places peptides at a supreme disadvantage compared to non-linear recognition motifs capable of forming 3-dimensional binding structures on antibodies and mimetic proteins like DARPins [289,290]. So far there only approaches to attempt to overcome this weakness in peptide design come in the form fiber-knob chimeras utilising large, non-adenoviral proteins such as single-chain antibodies. It is conceivable that modern de novo protein design techniques, in combination with biopanning, can be applied to the loop structure projecting from the adenoviral fiber-knob to enable the construction of highly avid 3-dimensional recognition sites against a pre-determined recognition epitope [291].

The final constraint on peptide-based approaches to adenoviral retargeting is the lack of availability of PTMs (Post Translational Modifications). As the adenoviral particle is assembled in the cytoplasm and is not trafficked through the Endoplasmic Reticulum (ER) or Golgi, precluding cysteine bond formation and glycosylation. Unless a method is found to facilitate in vivo post translational modification of adenoviral proteins without inhibiting virion assembly the peptide-based approach will remain limited to motifs which do not require PTMs.

## 6. Conclusions and Future Directions

Oncolytic virotherapies are progressing towards the forefront in the battle against cancer. In the two decades since the first trials of ONYX-015 began, mounting evidence has emerged that, with increasing knowledge of adenovirus biology and refinement of their mechanisms of action, oncolytic virotherapies are beginning to demonstrate efficacy, especially in combination with chemotherapies and immunotherapies. To further enhance their therapeutic index and safety, an ever-increasing wealth of virological information and technologies have been described to “tailor” viruses into bespoke, cancer fighting agents. In this review, we highlight two such areas.

Firstly, how manipulation of early viral genes can result in selective replication within the tumour microenvironment. Secondly, we comprehensively overview the abundance of knowledge around adenovirus structure and receptor interactions that can be exploited to tailor the viral tropism away from native infectious routes, into bespoke tumour targeted agents. It should be added, however, that additional levels of selectivity could and should be considered when designing virotherapies for clinical applications. For example, the use of tumour specific promoters to drive either replication or the expression of a therapeutic transgene, the use of microRNA (miR) binding sites to switch off replication or transgene expression in “off target” tissues, and the possibility of including a “safety switch” such as a Tet repressor or inducer, to switch off replication or transgene expression in the event of leaky infection, should all be considered. 

To add further complexity, careful consideration must be paid to which therapeutic transgene (or transgenes) should be encoded within the viral genome to maximise the anti-tumour efficacy of the oncolytic agent. The earliest studies focused on known immunostimulatory cytokines, such as GM-CSF and IL12, though leaky expression and secretion of these powerful cytokines from the tumour can potentially result in dose limiting toxicities. Recent advances extending our knowledge of how to best harness the immune system to mount an anticancer response through enhanced T-cell activation have shifted focus towards the use of virotherapies expressing immune checkpoint inhibitors and/or bispecific T-cell engagers (BiTEs), designed to be secreted from virally infected cells, and physically “bridge” the T-cell to tumour cells via a suitable bispecific fusion molecule (for example, a bi-specific anti CD3-anti CD19 molecule). It is no coincidence that the advances in the immunotherapy field have preceded and are now coinciding with the strides forward being made in the oncolytic field, providing an expanding armoury of agents for developing more sophisticated immunovirotherapies.

The evaluation of adenoviral vectors is complicated by the inadequacies of animal modeling in which to study them, as discussed by Baker et al. (2007) [292]. Rodents expressing xenografts enabling the study of tissues bearing human receptors (including cancers) are by necessity immunocompromised to prevent tissue rejection. However, this complicates the accurate evaluation of oncolytic virus interactions with components of the immune system, and activation of the immune system which (as discussed previously) is a major mode of therapeutic efficacy for oncolytic viruses [250]. Adenovirus is also incapable of replication within mouse cells precluding accurate evaluation of their lytic properties within mouse cells [293]. This has led to the popularization of hamster models for oncolytic adenovirus studies, which are semi-permissive to replication and remain immunocompetent whist supporting HaK tumours [294].

The situation is exacerbated in the context of evaluating CD46 engaging adenoviruses. Old World Monkeys express CD46 on erythrocytes, unlike humans, meaning that the virus may be sequestered in the blood [295]. In rodents CD46 expression is restricted to the testes, resulting in a restricted viral tissue tropism unlike that found in humans [296]. Taken together this makes both rodent and non-human primate models, the two main models used in drug development, of limited relevance to the development of oncolytic adenovirus.

While the limitations of the animal models may have hampered development, this has not hindered the translation of adenoviruses to the clinic. As well as the clinical trials already mentioned numerous other trials are, or have been, underway. Many of these are reviewed by Shaw and Suzuki, with more trials being added to clinicaltrials.gov every month [79].

We are constantly reminded that no two cancers are the same, each exhibiting their own unique signature of actively transforming modifications. It is therefore equally certain that no single immunovirotherapy will offer a “one size fits all” treatment for cancer. Individualised virotherapies will be required for personalised cancer treatments. We predict, therefore that the anti-cancer armoury of the future will contain a suite of immunovirotherapies, tailored at multiple levels of selectivity for virotherapy vector and serotype, to modified tropism, tumour specific expression driving replication, and a range of sophisticated immunotherapies and other transgenes. The momentum gathered by the fields of oncolytics and gene therapy now makes it all but certain that the next few decades will usher in the era of bespoke virotherapies.

## Figures and Tables

**Table 1:** Adenovirus receptor, the residues shown to facilitate receptor interactions, and demonstrated tropism ablating mutations. Prototype viral receptor refers to the adenoviral serotype and protein used in the receptor interaction study for which the binding residues and mutations are described. Named mutations are in bold with the mutations in brackets. amino acids are described with single letter code. Δ indicates a deletion mutation, while substitution mutations are described with the original residue letter codes preceding the residue numbers and then the respective substituted amino acid code.

Receptor	Prototype Viral Receptor	Receptor Binding Residues	Previously Demonstrated Tropism Aablating Mutations	References
CAR	Ad5—Fiber Protein knob	A406; S408; P409; R412; Y477; R481; L485; Y491	KO1 (SP408-409EA); KO2 (ΔVK441-44s); KO3 (R460E); KO4 (ΔGK509-510); KO5 (ΔGT538-539); KO8 (N468T); KO9 (V466H); KO10 (P505A); KO11 (Δ404-581 Whole region)	Jakubczak et al., 2001 [106]
			ΔTAYT (ΔTAYT489-492)	Roelvink et al., 1999 [107]
CD46	Ad35—Fiber Protein knob	F132; N133; T136; R169; M170; S172; N194; E192	F242; R279; S282	Wang et al., 2007 [108]
DSG2	Ad3—Fiber Protein Knob	N186; V189; S190; D261; F265; L292; L296; E299	N186D; V189G; S190P; D261N; F265L; L296R; E299V; ND186-261DN; ΔD261+L296R; NDL186-261-296DNR.	Wang et al., 2013 [109]
GD1a/Sialic acid	Ad37—Fiber Protein knob	Y308; Y312; P317; V322; K322	None reported	Nilsson et al., 2011 [110]
Blood Coagulation Factor X	Ad5—Hexon Protein HVR’s	HVR regions 3; and 7 (Individual residues not clearly defined).	Ad5HVR48 (Ad5 with the HVR’s of Ad48)	Waddington et al., 2008 [111]
			HVR5-BAP (71aa BAP (Biofilm Associated Protein) peptide insert)	Kalyuzhniy et al., 2008 [112]
			HVR5* (TE268-269AT); HVR7* (ITEL420-422-423-425GNSY); E451Q	Alba et al., 2009 [113]
HSPG	Ad5—Fiber Protein shaft	KKTK91-94	S* (KKTK91-94GAGA); KKTK91-94RGDK	Paolo et al., 2007, Kritz et al., 2007 [114,115]
Integrin	Ad5—Penton Protein	R340, G341, D342	RGE (D342E)	Bai et al., 1993 [116]
			EGD (R340E)	Henning et al., 2005 [117]
MARCO	Ad5—Hexon Protein	HVR1; implied but not conclusively determined	None reported	Stichling et al., [118]
